# A Continuum Model for the Effect of Dynamic Recrystallization on the Stress–Strain Response

**DOI:** 10.3390/ma11050867

**Published:** 2018-05-22

**Authors:** H. Kooiker, E. S. Perdahcıoğlu, A. H. van den Boogaard

**Affiliations:** 1 Philips HealthTech, Amstelplein 2, 1096 BC Amsterdam, The Netherlands; 2 Department of Nonlinear Solid Mechanics, University of Twente, Drienerlolaan 5, 7522 NB Enschede, The Netherlands; e.s.perdahcioglu@utwente.nl (E.S.P.); a.h.vandenboogaard@utwente.nl (A.H.v.d.B.)

**Keywords:** dynamic recrystallization, hot forming, austenitic stainless steel, high-strength low-alloy, strain rate, driving pressure, continuum modeling, dynamic stress

## Abstract

Austenitic Stainless Steels and High-Strength Low-Alloy (HSLA) steels show significant dynamic recovery and dynamic recrystallization (DRX) during hot forming. In order to design optimal and safe hot-formed products, a good understanding and constitutive description of the material behavior is vital. A new continuum model is presented and validated on a wide range of deformation conditions including high strain rate deformation. The model is presented in rate form to allow for the prediction of material behavior in transient process conditions. The proposed model is capable of accurately describing the stress–strain behavior of AISI 316LN in hot forming conditions, also the high strain rate DRX-induced softening observed during hot torsion of HSLA is accurately predicted. It is shown that the increase in recrystallization rate at high strain rates observed in experiments can be captured by including the elastic energy due to the dynamic stress in the driving pressure for recrystallization. Furthermore, the predicted resulting grain sizes follow the power-law dependence with steady state stress that is often reported in literature and the evolution during hot deformation shows the expected trend.

## 1. Introduction

In the mass-manufacturing industry there is an ongoing drive to improve product quality or reduce costs by reducing finishing steps. For e.g., automotive or consumer products made from austenitic stainless steel or High-Strength Low-Alloy steel (HSLA) there is a clear need to reduce springback, increase formability or decrease the amount of residual stress after forming [1,2]. To achieve these goals, one option is to apply a hot forming process where the material is processed at temperatures between 900 ${}^{\circ }$C and 1100 ${}^{\circ }$C. At these temperatures the yield stress of the material is considerably lowered. During hot forming, materials of low stacking fault energy experience concurrent hardening, dynamic recovery and dynamic recrystallization [3]. Dynamic recovery and dynamic recrystallization are softening processes of which recovery annihilates and rearranges dislocations into orderly arrays and recrystallization is the nucleation and growth of new grains that sweep the dislocated microstructure leaving it relatively dislocation free [4]. They cause extensive microstructural change and if the deformation and thermal conditions are not properly controlled during and after forming, the final part may lack the desired final strength [5].

Presently, modeling is an essential part of the design of new processes and products. Looking at hot forming, the biggest modeling challenge is dynamic recrystallization. Many types of dynamic recrystallization are described in literature, e.g., discrete dynamic recrystallization (DDRX), continuous dynamic recrystallization (CDRX) and post-dynamic recrystallization (PDRX) [4,6,7,8]. The first two describe the emergence of new grains in the microstructure during deformation, the latter describes the recrystallization processes after hot deformation including DRX. During the occurrence of CDRX no clear “nucleation” and “growth” stage occurs, rather the microstructure changes gradually resulting in a fine-grained structure of crystallites surrounded by high-angle grain boundaries [4]. This is phenomenologically different from DDRX where nucleation and growth are clearly visible. If a material will experience DDRX or CDRX depends on its stacking fault energy [7]. In this paper the focus is on the modeling of DDRX, henceforth simply dynamic recrystallization (DRX).

Several classes of constitutive models exist capable of describing DRX with varying degrees of accuracy and microstructural detail. These include multi-scale (Cellular Automata), phase field methods and continuum approaches. Cellular Automata and phase field models have the advantage of being able to spatially track complex inhomogeneous microstructures, whereas continuum models usually only account for the average properties of the microstructure. However, the currently available multi-scale models and phase field models do not show the desired agreement between experiment and model [9,10,11] and are too demanding in terms of computation time [12]. Especially for robust optimization of production processes (e.g., employing Design and Analysis of Computer Experiments (DACE)) this is still a major issue. Indeed for model-based process and product design continuum models are the preferred choice.

**Figure 1:**
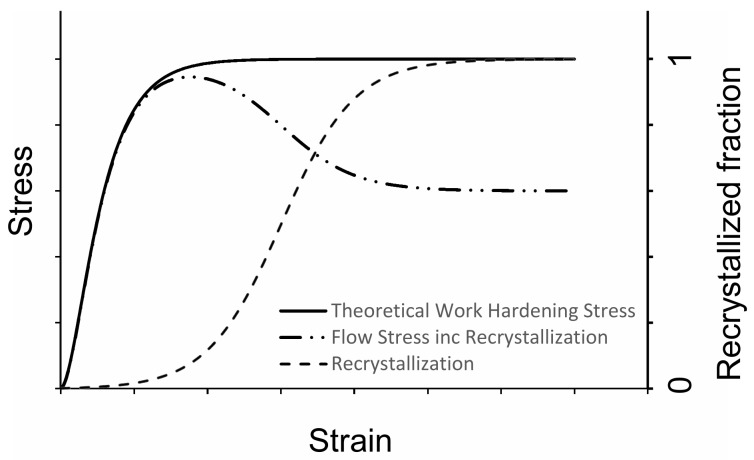
Avrami-based modeling of dynamic recrystallization.

The continuum models currently available can be divided into purely phenomenological/ mathematical representations of DRX and in more physically-based continuum representations of DRX. The phenomenological representations are usually based on the Avrami equation, splitting the yield stress into a work hardening stress, unaffected by recrystallization, and a softening stress with the transitional kinetics governed by an Avrami-type equation, see Figure 1. The softening stress is defined as the difference between the peak stress and steady state stress. Another class of phenomenological continuum descriptions of DRX is based on the Arrhenius equation and assume a relation between the flow stress and Zener-Holloman parameter *Z* [13]. To predict the evolution of the stress versus strain some constants are fitted with a 4th to 7th-order polynomial relation to strain, leading to highly nonlinear strain dependencies.

It has been shown that the aforementioned models are capable of capturing experimental characteristics of hot deformation tests like the peak strain, peak stress and steady state stress [14,15,16]. However they cannot be used for real process simulations because transient process conditions cannot be considered. Furthermore, they consider DRX as a “finite” strain-based process which finishes at the steady state. In reality, DRX isn’t finished at the steady state. At the steady state stress, the rate of dislocation generation and rate of recovery and recrystallization are in equilibrium [17] and recrystallization is ongoing with a constant rate.

The more physically-based continuum descriptions of DRX, model the process as time-based providing the opportunity for transient process modeling. Brown [5] takes recrystallization into account by looking at the influence of stored energy on grain and subgrain boundary mobility. It is assumed that recrystallization sweeps through the material, reducing the dislocation density to a low value. Based on the ideas introduced by Luton and Sellars [18] multiple recrystallization cycles may or may not overlap. When multiple recrystallization cycles sufficiently overlap, single peak recrystallization can be predicted, when recrystallization cycles are in slow succession, multiple peak recrystallization ensues. Another physically-based model, which is an extension of the work of Sandström and Lagneborg [19], is presented by Roucoules et al. [20]. They take the approach of defining a dislocation density distribution in the material, subsequently an evolution equation is presented for the distribution that accounts for hardening, recovery and recrystallization. They omit the split of dislocation density into a homogeneous dislocation density within subgrains and a dislocation density within subgrain walls as done by Sandström [19]. The essential feature of this model is the consideration of a distribution function to account for local variation of dislocation density driving recrystallization.

A significant amount of research has been presented on multi-grain representations of DRX. In these models the microstructure is represented by distinct grains, attributed with local characteristics like dislocation density, size and crystallographic orientation. The behavior of the grains is subsequently dictated by the macroscopic loading and the difference between its local properties and the averaged properties of all grains. Depending on local conditions new DRX-grains can be added to the assembly replacing part of a hardened grain and thus cause softening.

One of the first presentations of this type of model comes from Montheillet et al. [21]. Interestingly, it includes a geometrical softening effect dubbed boundary migration induced softening (BMIS), where the growing grain is softened by the increase of grain volume, i.e., the density of dislocations decreases when a grain increases in size without increasing the amount of dislocations. Based on the ideas of Montheillet et al. Bernard et al. have presented a similar multi-grain DRX-model. The novelty of this model lies in the split of the averaged medium in an averaged non-recrystallizing and averaged recrystallizing medium [22]. Grains can grow and diminish in size by interaction with the two media. Recently an update of the model has been presented by Beltran et al. to account for post dynamic recrystallization [23]. Another multi-grain model similar to that of Montheillet is presented by Cram et al. [24]. The novelty of this model is the addition of a local crystallographic orientation, influencing the boundary migration velocity of recrystallizing grains. Nucleation is coupled to a critical subgrain size and relations are presented for the growth and shrinkage of subgrains within a grain. Fan and Yang present a multi-grain model that describes recrystallization by modeling the volume consumption of moving mobile grain boundaries [25]. They track the nucleation, growth and impingement of mobile and immobile grain boundaries and also the conversion into each other. This model also takes into account that small nucleated grains experience less work-hardening compared to larger grains, by partitioning the macroscopic strain according to the iso-work assumption.

More recently Grätz [26], based on earlier numerical and experimental work of Roters et al. [27], presented a three-internal variable model capable of modeling recrystallization. In this model the work-hardening is based on the model for cell-forming materials proposed by Estrin et al. [28]. The macroscopic flow stress is determined by the weighted average of the flow stress of the cell and wall. The novelty of this model is the assumed nucleation mechanism, which is explicitly coupled to the thinning of cell-walls. Once a critical wall thickness is achieved, it is assumed that a cell is transformed to a subgrain with enhanced boundary mobility (compared to the cell) and thus the ability to grow or “bulge” into dislocation dense regions thereby recrystallizing the microstructure.

Industrial hot forming processes are usually characterized by high throughput and thus high strain rate deformations. Also, the range of deformation conditions within a single process-step can be quite large. With regards to the high strain rate hot forming behavior, it is observed that some steels (e.g., HSLA-steel) display significant stress softening at (very) high strain rates [24,29] (see Figure 3d). Here it can be seen that at a strain rate of 2 s^−1^ the steady state is reached after approximately 2.5 s and at a strain rate of 0.02 s^−1^ it is reached after approximately 100 s. Clearly the recrystallization rate at high strain rate is much larger than the rate at low strain rate, i.e., on average 1% versus 50% recrystallized volume per second. As of yet there is no clear consensus on the physical mechanisms causing the observed increase.

The recrystallization rate depends largely on the amount (nucleation) and growth of recrystallizing grains. Experimentally it has been confirmed that there is an almost linear relation between strain rate and nucleation rate [30,31,32] and some of the presented DRX-models already assume this strain rate dependence of nucleation-rate. Subsequently it was observed that, inspite of the increase in nucleation rate with strain rate, the recrystallization rate is still under-predicted at higher strain rates. Therefore models are fitted separately for each strain rate condition, leading to the conclusion that the grain boundary mobility must be enhanced at higher strain rates in order to increase the grain boundary velocity and obtain agreement between model and experiment [21,22,33], even though no clear physical explanation is given for this dependence [34]. The grain boundary velocity is the product of the driving pressure and mobility (see Section 2.2.4). In this work it will be assumed that increased grain boundary velocity at higher strain rate is not due to an increase in grain boundary mobility, instead it is proposed that at higher strain rate the driving pressure for grain boundary migration is increased. Indeed at a higher strain rate, due to reduced time for recovery, there is a larger driving pressure for migration because of increased dislocation density. However it will be shown that the driving pressure should be supplemented with an additional mechanical driving pressure due to the dynamic stress. The approach assumes that mobility is an intrinsic material property unaffected by the applied strain rate, even-though it is recognized that the thermally activated nature of the boundary movement does not preclude an effect of strain rate on mobility.

In general, the previously discussed physically-based models are quite good at representing some of the key features of DRX like resulting grain size, the initiation of DRX and the transition from single to multiple peak behavior. However they are currently not ready for predicting the behavior of industrial hot forming processes in full-scale Finite Element (FE) process simulations, because they are usually validated on a limited amount of stress–strain curves within a narrow range of deformation conditions [8,22,23,26,34]. Furthermore, they often do not consider the behavior at high-strain rate deformation conditions at all [22,23,25,26]. Some authors did attempt to validate their model at high strain rate deformation conditions. One of these is the model presented by Cram et al. It is one of the more “complete” multi-grain models taking a wide range of local microstructural-characteristics into account. Despite of this, it under predicts the recrystallization softening at higher strain rate [32]. The same holds for the model presented by Favre et al. which also underpredicts high strain rate DRX-induced softening [33].

Despite the significant amount of available models, there is clearly room for improvement on the agreement between stress–strain behavior, especially at high strain rate deformation. The goal is to have a model that accounts for the effect of DRX on the stress–strain behavior for a wide range of processing conditions, including high strain rate deformation. Eventually the model must be suitable for FE simulations of hot forming processes and optimizations thereof and therefore computational efficiency is very important. Although the multi-grain representations have the benefit of being able to represent many of the key characteristics of DRX, they are not considered since ultimately, they do not meet the demands towards computational efficiency, especially during grain refinement, where an exceeding amount of grains need to be accounted for [32]. To be able to predict arbitrary loading paths the model is presented in rate form. Calibration and validation is performed on the experiments presented by Zhang et al. [16] which pertain hot compression tests of AISI316LN for a wide range of temperatures and strain rates, furthermore the hot torsion experiments on HSLA-steel presented by Roucoules et al. are considered which show significant stress-softening at high strain rates [29] to validate the proposed addition to the driving pressure for grain boundary migration.

## 2. Continuum Mechanical Model for Hot Forming

The description of the continuum mechanical model is split in two sections, the first section describes the formulation for the yield stress including the effect of hardening and dynamic recovery, the second section describes the way in which the effect of DRX on the stress–strain curves is accounted for.

### 2.1. Yield Stress Description

Laasraoui et al. have shown that the Bergström equation can successfully describe hardening and recovery of steels at elevated temperatures and a wide range of strain rates [35]. The Bergström model splits the deformation resistance in three parts, i.e., yield stress, in three parts [36,37]:

(1) $$\sigma_{y}=\sigma_{i}+\sigma_{w}+\sigma^{*}$$

Here $\sigma_{i}$ is the strain and strain rate independent frictional stress due to the lattice (Peierls stress) [38], $\sigma^{*}$ is a dynamic stress which is rate and temperature dependent and $\sigma_{w}$ is the work hardening stress which is governed by the statistical density of immobile dislocations, henceforth ρ. The relation between the dislocation density and the work hardening stress is given by the Taylor equation:

(2) $$\sigma_{w}=\alpha \mu bM\sqrt{\rho }$$

In which α, μ, *b* and *M* are a material constant, temperature dependent shear modulus, the length of the Burgers vector and the Taylor orientation factor respectively. The temperature dependence of the frictional stress $\sigma_{i}$ is assumed similar to the temperature dependence of the shear modulus and Equation (1) is therefore rewritten to:

(3) $$\sigma_{y}=\mu \left(T\right)({\sigma_{i}}_{0}+\alpha \mu_{ref}bM\sqrt{\rho })+\sigma^{*}$$

where μ(T) is the temperature dependence of the shear modulus with respect to a reference value $\mu_{ref}$ and is modeled as a simple linear function of temperature:

(4) $$\mu \left(T\right)=\frac{\mu_{ref}-c_{\mu }T}{\mu_{ref}}$$

The strain rate and temperature dependent dynamic stress is given by:

(5) $$\sigma^{*}=c_{ds}\exp\left(\frac{Q_{ds}}{RT}\right){\left(\ln\frac{\dot{\varepsilon }}{\dot{\varepsilon_{0}}}\right)}^{n_{ds}}$$

where $\dot{\varepsilon_{0}}$ can be taken as the lowest strain rate in the validation set.

#### 2.1.1. Work Hardening and Dynamic Recovery

To describe the evolution of the work hardening stress a supplementary equation is needed to predict the evolution of dislocation density with strain. The well known Bergström equation can describe the storage and loss of immobile dislocation density under the influence of strain successfully over a range of temperatures and strain rates [38,39,40].

(6) $$\frac{d\rho }{d\varepsilon }={\frac{d\rho }{d\varepsilon }}^{+}+{\frac{d\rho }{d\varepsilon }}^{-}=h\sqrt{\rho }-f\rho$$

In which $h\sqrt{\rho }$ describes the hardening by storage of dislocations and it is inversely proportional to the mean free path of dislocations, which decreases when the dislocation density increases corresponding with the principle of similitude. It is known that annealed austenitic stainless steels tend to form subgrains (dislocation cells) after some deformation and that this has a significant effect on the mean free path [41], therefore the proportionality factor *h* evolves with strain as well, this is explained further in Section 2.1.2. The dynamic recovery term *f* is dependent on temperature and strain rate due to its thermally activated nature, this can be modeled by employing the Zener-Holloman parameter *Z* [39], which incorporates the combined effect of strain rate and temperature:

(7) $$f=f_{0}+c_{f}Z^{n_{f}}$$

#### 2.1.2. Evolution of Mean Free Path on Hardening Behavior

Experimental results of Angella [41] show that the mean free path of AISI 316L does not obey the principle of similitude during hot forming. Transmission Election Microscopy (TEM) results show that the material starts out with homogeneously spaced dislocations which, upon straining, arrange into a cell pattern. The rearrangement from homogeneous spacing to cell pattern is accompanied by an increase in mean free path with strain and consequently a decrease in hardening rate. This is due to the relatively low dislocation density in cell-interiors with respect to cell-walls, decreasing the rate at which mobile dislocations are converted to immobile dislocations. Several authors have also noted that similitude does not always hold, especially at higher temperatures [37,40].

To model the transition from homogeneous dislocations to cells Angella assumes that the proportionality constant of the mean free path is a state variable (next to the dislocation density), which evolves from an initial value to a saturation value. It is shown that this model is capable of better capturing the small strain (up to 0.15 true strain) behavior during hot forming of these materials [41,42]. The proposed evolution equation for the mean free path proportionality constant *h* is:

(8) $$\frac{dh}{d\varepsilon }=\frac{K}{\sqrt{\rho }}\left(h_{s}-h\right)$$

where $h_{s}$ is the saturation value and *K* governs the rate at which cells are formed, i.e., the transition from $h_{0}$ to $h_{s}$ ($h_{0}>h_{sat}$). The transition rate from homogeneously distributed dislocations to cells is however strain rate and temperature dependent and therefore *K* is made dependent on the Zener-Holloman parameter, similar to the strain rate and temperature dependence of dynamic recovery (Equation (7)) [42].

### 2.2. Effect of Dynamic Recrystallization on the Yield Stress

During dynamic recrystallization new relatively dislocation-free grains nucleate and grow, thereby overtaking the dislocated microstructure. During the lifetime of such a grain it can be in four phases:

- *Nucleation* Depending on the prevailing mechanism, i.e., grain boundary bulging, twinning, or nucleation from subgrains, a new dislocation-poor grain nucleates into the dislocated microstructure.
- *Growth* Driven by the difference in dislocation density within the new grain and its surroundings the grain boundary migrates increasing the size of the new grain at the expense of dislocation-rich surroundings
- *Growth stagnation* Concurrent hardening, due to ongoing deformation during the growth-phase, increases the dislocation density within the grain to be almost equal to the dislocation density of the surroundings effectively halting growth
- *Hardening* Continued deformation increases the dislocation density within the grain such that itself becomes a site for “new” nucleation.

During the course of hot deformation, depending on the hardening, new grains continuously nucleate. This leads to an evolving distribution of recrystallizing grains, of which the grains are all in one of the aforementioned phases. Growth acts to shift the distribution to larger grain sizes, nucleation removes the larger-sized grains from the distribution, adds smaller-sized grains to the distribution and increases the amount of recrystallizing grains.

**Figure 2:**
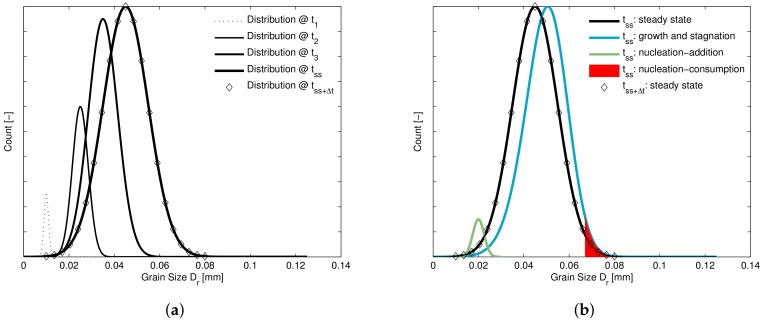
(**a**) Change of the distribution of recrystallizing grains from the onset of DRX to the steady state. (**b**) Detail of the effect of several mechanisms on the distribution at the steady state showing that the net effect is zero, leading to a constant distribution.

At the steady state stress the rate of nucleation, leading to disappearance of larger-sized grains and addition of small grains to the distribution, is in equilibrium with the growth of the growth-capable grains. This implies that, at the steady state stress, the amount of grains partaking in recrystallization and the average size of these grains is constant, the concept is further explained in Figure 2.

The essence of the proposed recrystallization model is that it is assumed that the effect of recrystallization on the stress–strain behavior can be described by looking at the softening introduced by the volume consumption of dislocation dense material by the distribution of recrystallizing grains. Subsequently, it is assumed that the distribution of recrystallizing grains can be represented by the amount of recrystallizing grains and the average size of these grains. The latter assumption is made for reasons of computational efficiency, avoiding the tracking of nucleation and current size of a large amount of grains.

#### 2.2.1. Recrystallization Rate

The recrystallization rate, i.e., the rate at which the microstructure is being replaced by new dislocation-poor grains, can be described by the rate of volume consumption of the distribution of recrystallizing grains. At this point it is assumed that the recrystallizing grains are spherical and thus the relative volume consumption can be described by:

(9) $$\dot{R}=\frac{1}{6}\pi \dot{N}{\bar{D}}^{3}+\pi N{\bar{D}}^{2}\bar{v}$$

where *N* is the amount of grains partaking in recrystallization per unit volume, $\bar{D}$ is the average size of the recrystallizing grains and $\bar{v}$ is the average grain boundary velocity. To complete the model for the effect of DRX on the stress-strain behavior four key relations are needed, i.e., an evolution equation for the average size of the distribution of recrystallizing grains, an evolution equation for the amount of grains partaking in recrystallization, a relation for the average grain boundary velocity and a relation coupling the effect of recrystallization to the Bergström equation.

#### 2.2.2. The Amount of Recrystallizing Grains

In the classical thermodynamical treatment of nucleation of a spherical grain, a “critical radius” of the nucleus is determined by the balance between consumed dislocation density and “old” grain boundary versus the creation of new grain boundary. In a treatment by Hutchinson [3] it was established that, in general, DRX-nucleation does not seem to be captured by this approach because of the high interfacial energy of high angle grain boundaries and low stored energy of deformation, which cannot realistically be overcome by thermal fluctuations alone. As a result, new physical mechanisms of nucleation were proposed and different views evolved in part due to the experimental difficulty associated with in situ observation of nucleation [8,31]. Three commonly accepted possible mechanisms are the bulging mechanism based on the proposal of Bailey and Hirsch [43], the formation of nuclei from special subgrains [44] and nucleation resulting from migrating twin boundaries [8,45,46,47].

In this research it is the objective to end-up with a predictive model for the stress–strain behavior during hot forming. Special attention is given to the necessity to account for the effect of stress on the driving pressure for grain boundary migration. Therefore it is chosen to implement a nucleation criterion that is flexible enough to describe the experimentally observed temperature and strain rate dependence of nucleation and to account for any physical type of nucleation, this is also done by e.g., Montheillet et al. [21]. This approach allows to show clearly the necessity of introducing an enhancement of grain boundary migration at higher strain rate. Several of these flexible phenomenological nucleation relations exist, the one used in this work is based on the one used by Hallberg [48].

The proposed description of DRX does not require a classical nucleation rate, it requires the rate of change of amount of grains partaking in recrystallization, i.e., $\dot{N}$. The amount of grains that are recrystallizing cannot increase indefinitely. The recrystallization rate is defined as the fractional replacement of deformed volume per unit volume. If unit volume is considered, then the total volume of the assembly of recrystallizing grains cannot exceed this volume (including nuclei, growing grains, growth-stagnant grains and deformed grains). Therefore the maximum volume of the assembly of the recrystallizing grains provides the constraint on both the amount and average size. The rate of change of the amount of recrystallizing grains is described by:

(10) $$\dot{N}=c_{n}{\dot{\varepsilon }}^{p}\exp(\frac{-Q_{n}}{RT})\left(1-V_{r}\right)$$

where $c_{n}$ is a constant, $Q_{n}$ is the activation energy for nucleation and *p* is a constant reflecting the strain rate dependency of the nucleation rate (taken as 1) and $\left(1-V_{r}\right)$ is the constraint on nucleation. Similar to Brown [5] and consistent with the model observations of Roucoules et al. [20], no explicit criterion for the onset of nucleation is assumed (like a critical dislocation density). The nucleation activation energy is taken as constant, however it is likely to be affected by local stored energy. The volume of the recrystallizing grains is defined as:

(11) $$Vr=\frac{1}{6}\pi N{\bar{D}}^{3}$$

#### 2.2.3. The Average Size of the Recrystallizing Grains

The average size of the recrystallizing grains is determined by integrating the rate of change of $\bar{D}$. Therefore a relation is needed to describe the rate of change of $\bar{D}$, i.e., $\dot{\bar{D}}$, which is governed by nucleation, growth and, similar to nucleation, cannot grow indefinitely. The change in the average grain size due to nucleation can be modeled by proportionally lowering $\bar{D}$ towards the size of the recrystallization nucleus ${\bar{D}}_{0}$. Growth of the average recrystallizing grain size is determined by the average grain boundary velocity $\bar{v}$. In the current model it assumed that the growth of the average size is halted when the volume of the assembly of recrystallizing grains ($V_{r}$) approaches unit volume. Obviously growth also stops when the average grain boundary velocity is zero.

(12) $$\dot{\bar{D}}=2\bar{v}\left(1-V_{r}\right)-\left(\bar{D}-{\bar{D}}_{0}\right)\frac{1}{N}\dot{N}$$

It is important to note that the parameters *N* and $\bar{D}$ describe the amount of recrystallizing grains and their average size. They do not describe the growth and nucleation of single grains. If the rate of change of either *N* or $\bar{D}$ is zero (e.g., at the steady state) this does not mean that there is no growth and no nucleation. Indeed the grains that make up the distribution still grow, new ones are continuously added by nucleation and older-hardened grains are continuously being replaced by nucleation and growth of less-hardened grains, however at this point these effects do not change the distribution anymore leading to a constant recrystallization rate.

#### 2.2.4. Average Grain Boundary Velocity and Driving Pressure

The average boundary migration velocity is derived from the relation between the pressure on the grain boundaries and its subsequent migration rate [4]:

(13) $$\bar{v}=mP$$

Here *m* is the grain boundary mobility, *P* is the driving pressure. The grain boundary mobility is temperature dependent and is given by the Stokes-Einstein relationship [4].

(14) $$m=\frac{m_{0}}{T}\exp(\frac{-Q_{m}}{RT})$$

In which $Q_{m}$ is the apparent activation energy for boundary migration. As stated in the introduction some materials show a significant amount of stress-softening at higher strain rates and increased nucleation rate alone cannot account for this. The approach taken by others is to link the increased grain boundary migration to an increase in grain boundary mobility. They effectively fit stress–strain curves at increasing strain rate and show that the best fitting results are obtained when mobility is significantly increased.

However it is known that stress can have a significant effect on recrystallization, e.g., Toda-Caraballo et al. [49] found that (residual) stress can have a large influence on recrystallization behavior. Specifically Senkov, Jonas and Froes compared the driving pressure for grain boundary migration due to applied stress, with that of the dislocation energy and concluded that the former is (much) higher than the driving pressure due to dislocation energy only. Some predictions were made for the steady state behavior in hot torsion tests showing a good match between model and experiment [50].

Recrystallization is a process with which the material lowers its Gibbs free energy by nucleating new dislocation free grains. Kocks et al. point out that the change in flow stress by a change in strain rate (i.e., dynamic stress), is proportional to the concentration of obstacles to glide [51]. In this case the obstacles to glide are the dislocations tangles caused by workhardening and as such when recrystallization lowers the dislocation (obstacle) density, the potential for dynamic stress build-up is also lowered. Seeing that the dynamic stress adds to the internal energy of the system it is, therefore, reasonable to assume that the system experiences an additional driving pressure stemming from the elastic energy of the dynamic stress. In the results section it will be shown that the proposed addition of a dynamic component to the driving pressure, is essential in capturing the rate dependent behavior of dynamic recrystallization seen in the experiments. The driving pressure is modeled by:

(15) $$P=\frac{1}{2}\mu b^{2}\rho +a_{d}\frac{{\sigma^{*}}^{2}}{\mu }$$

The term $\frac{{\sigma^{*}}^{2}}{\mu }$ represents the strain energy density imparted on the material by the dynamic stress. Note that the driving pressure term due to the dislocations similarly represents a strain energy density (∼$\frac{\sigma_{w}^{2}}{\mu }$). It must be noted that Equation (15) is similar to the one proposed by Senkov et al., however they considered the steady state stress only.

Multi-scale (grain) models have the ability to track local dislocation density, this has the advantage of letting the migration be dictated by the difference between the dislocation density inside the growing grain and the surroundings. However in the current work it is not the local grain boundary velocity that is being modeled, it is the average grain boundary velocity, which is assumed to be correlated with the average dislocation density, i.e., if the average dislocation density in the material is high, there must also exist high dislocation density gradients.

#### 2.2.5. Coupling of DRX to the Bergström Equation

The presented equations for DRX have to be coupled to the Bergström equation to be able to account for the softening. This is accomplished by adding an additional term to Equation (6):

(16) $$d\rho =\left(h\sqrt{\rho }-f\rho \right)d\varepsilon -\dot{R}\left(\rho -\rho_{0}\right)dt$$

where $\dot{R}$ is the recrystallization rate defined as the fractional replacement of deformed volume per unit volume. The recrystallized volume overtakes regions of high dislocation density ρ and leaves it with a low dislocation density $\rho_{0}$. As was stated in the introduction, recrystallization is modeled by its rate, thereby circumventing a finite recrystallization (i.e., stopping at 100%).

## 3. Model Results and Discussion

**Figure 3:**
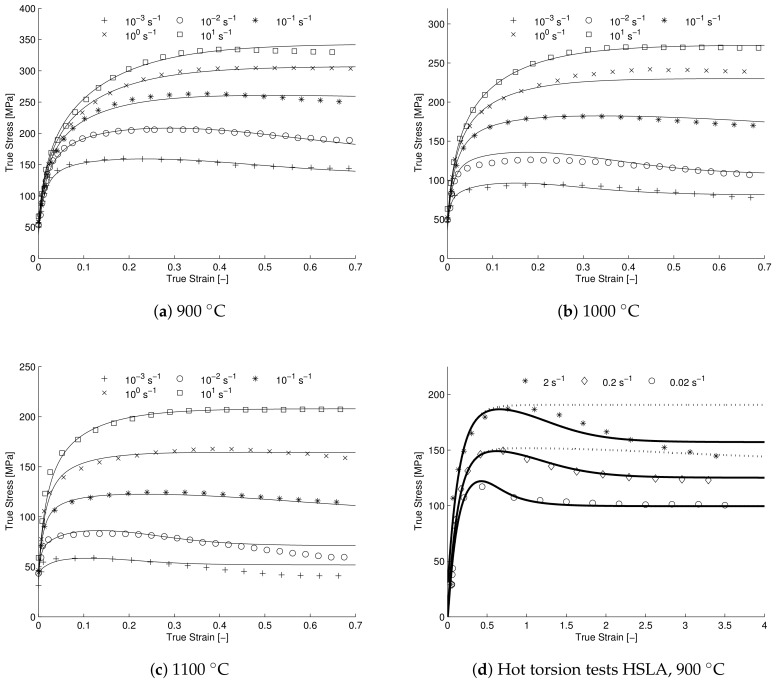
(**a**–**c**) Model validation for AISI 316LN for strain rates ranging from $10^{-3}$ s${}^{-1}$–$10^{1}$ s${}^{-1}$ and temperatures ranging from 900 ${}^{\circ }$C–1100 ${}^{\circ }$C, model results shown in solid line. (**d**) Model validation for HSLA-steel for strain rates ranging from 0.02 s${}^{-1}$–2 s${}^{-1}$ and a temperature of 900 ${}^{\circ }$C.

The proposed model was calibrated on two types steel, with the aim of validating the model predictions for wide ranging temperature and strain rate combinations and specifically for significant high strain rate DRX-induced softening. The first validation is shown in Figure 3a–c pertaining to the hot compression experiments performed by Zhang et al. on a Gleeble-1500D at a wide range of strain rates and temperatures. The second validation is performed on the hot torsion tests performed by Roucoules et al. on HSLA-steel [29] and is shown in Figure 3d including the model result with and without the proposed addition to the driving pressure.

For AISI 316LN the model comprises in total 25 parameters, of which 5 are fixed material parameters and two were selected (α, *b*, *M*, $\mu_{ref}$ and $c_{\mu }$ versus $D_{0}$ and *p* respectively), the remainder were fitted by least squares optimization to the hot compression data. First the parameters pertaining to the hardening and recovery were fitted to the initial part of the stress–strain curves, next the DRX-related parameters were fitted to the entire stress-strain curve, by means of least squares optimization. Note that only 5 parameters are used to describe the strain rate and temperature dependent dynamic recrystallization behavior. The model parameters and estimates are shown in Table 1. During the optimization of the parameters it was found that the best fit could be obtained by excluding $\sigma_{i}$ from the model. For the validation of the high strain rate DRX-induced softening of HSLA-steel, it was possible to omit the temperature dependence of several mechanisms (because temperature variation was not considered for this steel) leading to a reduced model. The model parameters are shown in Table 2, note that not all parameters are exactly comparable to the parameters shown in Table 1. Some of the parameters are a combination of the thermal activation at 900 ${}^{\circ }$C and the constant, e.g., $c_{f}$, $m_{0}$, $c_{ds}$ and $c_{n}$, furthermore the transition parameter *K* from Equation (8) is not needed for the prediction of HSLA-steel and therefore also omitted. Lastly in Table 3 the driving pressure due to dislocation density and the dynamic stress stress is shown.

In Figure 3, it can be seen that there is good agreement between model and experimental data for both AISI 316LN and HSLA-steel. Some discrepancies remain, e.g., for AISI 316LN the softening seems to be slightly under-predicted at the highest temperature lowest strain rate combination. It is believed that at these deformation conditions, mechanisms other than DRX may start to play a role in determining the constitutive behavior. Indeed a normal annealing temperature-cycle for austenitic stainless steel consists of holding the material at a temperature of roughly 1080 ${}^{\circ }$C for approximately 5 to 10 min [52]. At the lowest strain rate and highest temperature the experimental conditions are thus more than sufficient for annealing to occur, the difference being the ongoing deformation. It is, therefore, realistic to assume that normal or abnormal grain-growth processes are concurrent to the deformation induced DRX-processes and could be affecting the softening.

**Figure 4:**
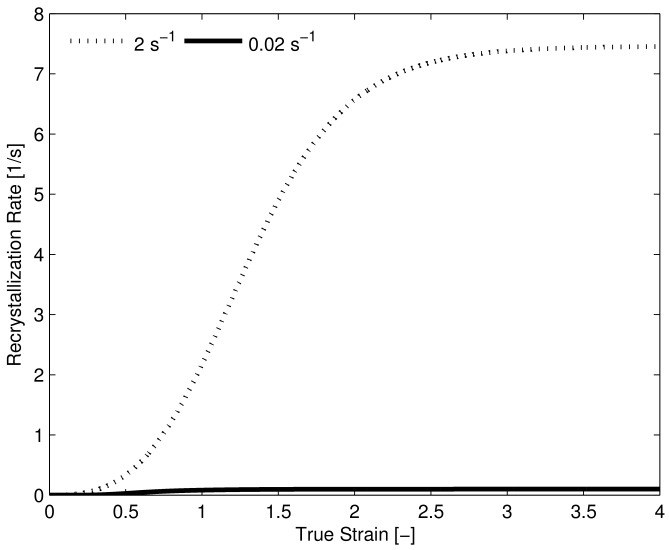
Predicted recrystallization rate HSLA-steel for strain rates 0.02 s${}^{-1}$ and 2 s${}^{-1}$, the maximum recrystallization rate at the higher-strain rate is approximately 74 times that of the lower strain rate.

For the HSLA-steel the DRX-induced softening, although very pronounced, is still slightly under-predicted at the highest strain rate. It is possible that the current DRX-model is missing some physical mechanism with which DRX can be accelerated. However another (likely) possibility could be adiabatic heating at the highest strain rate, causing the yield stress to drop due to an increase in temperature during the experiment. In Figure 3d the model prediction shows clearly that the recrystallization rate is severely under-predicted if only the increase in dislocation density is considered. In Table 3 it can be seen that the difference in dislocation density between the lowest and highest strain rate is only 50% which is not enough to significantly increase the recrystallization rate at this strain rate. Clearly the proposed addition of the elastic energy density caused by the dynamic stress to the driving pressure aids the agreement between the observed recrystallization characteristics of experiment and model. Furthermore, the predicted driving pressure contribution due to the dynamic stress, is in-line with the values found by Senkov et al. [50]. Note that an extremely high recrystallization rate is predicted by the model, this point is further highlighted by Figure 4 where the predicted recrystallization rate ($\frac{dR}{dt}$) is plotted versus strain for the lowest and highest strain rate experiment, showing a 74-fold increase in predicted recrystallization rate between the two deformation conditions.

### Grain Size Prediction

Inherent to the prediction of the effect of recrystallization, is the prediction of an average recrystallizing grain size throughout the hot deformation process. Although not the goal of the present model, it is still possible to check if the current model is predicting recrystallized grain sizes that are in agreement with the expected trends. Several authors have presented experimentally measured grain sizes at the end of hot deformation tests and it is often found that there is a power-law relation between the steady state stress and the steady state grain size for a wide range of materials [31,53,54,55].

(17) $$1<(\frac{\sigma_{ss}}{\mu })(\frac{{\bar{D}}_{ss}}{b}{)}^{2/3}<10$$

**Figure 5:**
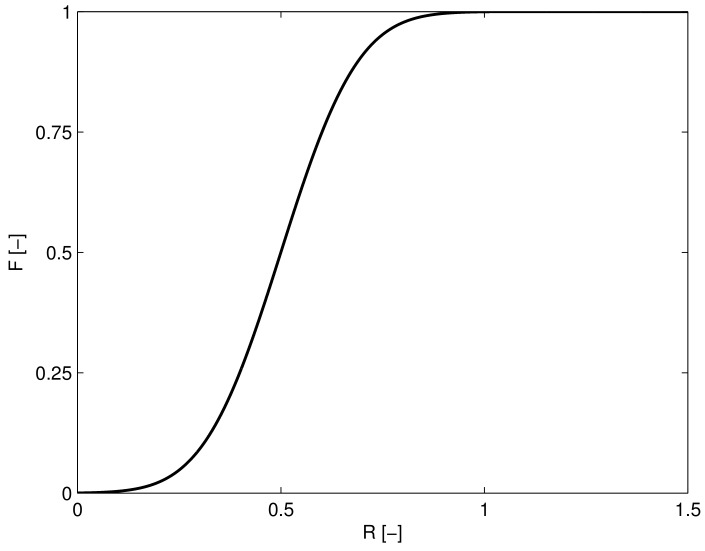
Transition parameter *F* depending on the extent of recrystallization *R*.

It is important to note that care should be taken when comparing the average recrystallizing grain size $\bar{D}$ predicted by the proposed model with these experimental results. At the start of dynamic recrystallization the material will still have its original microstructure e.g. an equiaxed microstructure of grain size $D_{i}$. Depending on the progress of recrystallization the microstructure will start to resemble this average recrystallizing grain size $\bar{D}$. It is, therefore, proposed to model the transition from the initial grain size $D_{i}$ to $\bar{D}$ with a simple sigmoid function which depends on the state of recrystallization *R*.

(18) $$D_{a}=\bar{D}F\left(R\right)+D_{i}(1-F\left(R\right))$$

where F(R) is the generalized normal cumulative distribution with mean 0.5 and spread 0.15 evaluated at *R*, see Figure 5. This implementation ensures that the grain size is completely represented by the average recrystallizing grain size when the entire material has recrystallized (R>1). Note that the quantity $D_{a}$ can be determined by post-processing, it does not directly influence the coupled differential equations that determine the constitutive response during hot forming.

**Figure 6:**
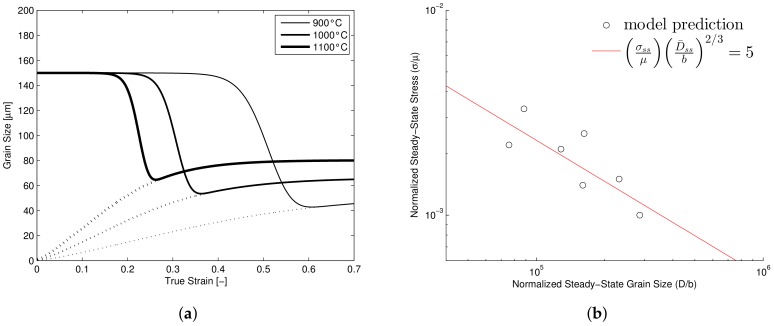
(**a**) Grain size evolution for three temperatures at strain rate $10^{-3}$ s${}^{-1}$ in which the solid lines represent $D_{a}$ and the dotted lines represent $\bar{D}$. (**b**) Predicted relation between normalized steady state stress and grain size.

In Figure 6a the predicted evolution of the average grain size for three different temperatures is shown. The model predicts an increased grain size with increasing temperature, in line with the increase of grain boundary mobility at high temperature. Using the proposed transition between the initial grain size and the recrystallizing grain size, results in a grain size evolution which is very similar to the one predicted by the model presented by Galindo-Nava et al. [56]. For the AISI 316LN model the steady state grain sizes according to Equation (18) were collected and it is shown that the model adheres to the power-law relation within the suggested bounds, see Figure 6b providing further confirmation that the model, at least qualitatively, captures the appropriate trends.

## 4. Conclusions

A new continuum model was proposed to account for the effect of DRX on the stress–strain evolution during hot forming. It was shown that:

1. The model is capable of accurately describing the stress–strain behavior of AISI 316LN over a wide range of temperatures and strain rates.
2. The high strain rate DRX-induced softening seen during hot torsion of HSLA-steel is appropriately captured.
3. Grain boundary migration velocity at higher strain rates can be predicted by adding the elastic energy, imparted by the applied dynamic stress, to the driving pressure thereby accurately predicting the extreme differences in recrystallization rate at high strain rate.
4. The predicted average steady state grain size is in good agreement with the expected power-law relation with the steady state stress.

Some discrepancies can still be seen at high strain of the highest temperature lowest strain rate combinations of AISI 316LN where the softening in the experiments exceeds that of the model. It is believed that this may be due to concurrent grain growth. Also the softening of HSLA-steel at the highest strain rate is slightly underestimated which may be due to adiabatic heating during the experiment.

## 5. Future Work

As future work the authors would like to validate the model further by adding grain size measurements (initial and steady state) to the validation set. Also inter-pass holding is of interest and possible predictions for post-dynamic recrystallization could be added by introducing a static recrystallization mechanism to the model.

## Figures and Tables

**Table 1:** Model parameter values. Fitted by least squares optimization to the hot compression stress–strain data of Zhang et al. [16].

α	0.5	$h_{0}$	9.05 × 10^5^ mm^−1^	$K_{0}$	3.05 × 10^5^ mm^−1^	$m_{0}$	5.69 × 10^9^ mm^4^K/Js
*b*	2.8 × 10^−7^ mm	$h_{s}$	1.90 × 10^5^ mm^−1^	$c_{k}$	2.59 × 10^12^ mm^−1^	$Q_{m}$	219 kJ/mol
*M*	3	*Q*	411 kJ/mol	$n_{k}$	−0.51	$c_{n}$	3.90 × 10^9^ s^−1^mm^−3^
$\mu_{ref}$	8.7 × 10^4^ N/mm^2^	$f_{0}$	11. 22	$c_{ds}$	1.5 × 10^−3^ MPa	$Q_{n}$	95.7 kJ/mol
$c_{\mu }$	26.3 MPa/K	$c_{f}$	3.1 × 10^4^	$n_{ds}$	0.81	$a_{d}$	5.16 × 10^2^
$\rho_{0}$	2.55 × 10^6^ mm^−2^	$n_{f}$	−0.2	$Q_{ds}$	74.0 kJ/mol	$D_{0}$	1× 10^−3^ mm

**Table 2:** Model parameter values. Fitted by least squares optimization to the hot torsion test data of Roucoules et al. [29].

α	0.5	$\rho_{0}$	6.27 × 10^5^ mm^−2^	$n_{f}$	−0.08	$c_{n}$	2.86 × 10^5^ s^−1^mm^−3^
*b*	2.8 × 10^−7^ mm	$h_{s}$	1.74 × 10^5^ mm^−1^	$c_{ds}$	1.69 MPa	$a_{d}$	1.69 × 10^2^
*M*	3	$f_{0}$	1.5	$n_{d}$	1.92		
μ	7.97 × 10^4^ N/mm^2^	$c_{f}$	12.53	$m_{0}$	2.53 mm^4^/Js		

**Table 3:** Comparison between the driving pressure contributions from dislocation density and dynamic stress.

$\dot{\varepsilon }$ [s^−1^]	P(ρ) [MPa]	P($\sigma^{*}$) [MPa]	$\sigma^{*}$ [MPa]
0.02	0.19	0	0
0.2	0.25	0.67	8.34
2	0.30	9.47	31.48

## Abbreviations

The following abbreviations are used in this manuscript:

AISI	American Iron and Steel Institute
HSLA	High-Strength Low-Alloy
DRX	Dynamic Recrystallization
DDRX	Discrete Dynamic Recrystallization
CDRX	Continuous Dynamic Recrystallization
PDRX	Post Dynamic Recrystallization
FEM	Finite Element Method
DACE	Design and Analysis of Computer Experiments
TEM	Transmission Electron Microscopy
BMIS	Boundary Migration Induced Softening
